# Response to “Comment on ‘Effects of Atrazine on Estrogen Receptor α– and G Protein–Coupled Receptor 30–Mediated Signaling and Proliferation in Cancer Cells and Cancer-Associated Fibroblasts’”

**DOI:** 10.1289/ehp.1611288

**Published:** 2016-04-01

**Authors:** Rosamaria Lappano, Marco Pupo, Marcello Maggiolini

**Affiliations:** 1 Department of Pharmacy, Health and Nutritional Sciences, University of Calabria, Rende, Italy; 2 Department of Cellular Biology, University of Geneva, Geneva, Switzerland

In their letter to the editor, Chevalier et al. wrote that a single chemical can elicit both agonist and antagonist activity through a specific receptor in different types of cancer cells, while two different chemicals can elicit opposite biological effects through the same receptor in the same cancer cell type, with the disparity lying in the receptor’s activation and in whether the action is primarily genomic versus nongenomic. Chevalier et al. also raised interesting issues regarding the level of exposure to chemicals and the cell context–dependent involvement of different transduction pathways by one chemical through the same receptor. These issues are complicated by the fact that the cumulative and final action may be triggered by a mixture of pollutants, in which the signal(s) that is/are activated by one or more compounds depend on the nature of the stimulus, its length, and the cell-related gene profile that characterizes a distinct cell context (Diamanti-Kandarakis et al. 2009).

For instance, as Chevalier et al. mention, 17α-estradiol (E_2_) inhibited cell proliferation through estrogen receptor (ER) β in human testicular seminoma–derived JKT-1 cells and seminoma tumors, whereas bisphenol A (BPA) promoted growth responses through G protein–coupled estrogen receptor (GPER) and the activation of protein kinase A and protein kinase G transduction pathways, but not through extracellular-signal-regulated kinase (ERK) signaling (Bouskine et al. 2009). Nevertheless, the epidermal growth factor receptor/ERK transduction pathway mediated the proliferation of spermatogonial GC-1 cells induced by BPA (Sheng and Zhu 2011) in accordance with our results obtained in breast cancer cells and cancer-associated fibroblasts (Pupo et al. 2012). Chevalier et al. also cited findings on atrazine action through GPER toward the suppression of JKT-1 cell proliferation (Fénichel et al. 2013). However, as we demonstrated in our study, GPER was able, on its own, to trigger the stimulatory effects induced by atrazine in ERα-negative breast cancer cells and cancer-associated fibroblasts, whereas atrazine stimulation involved a functional cooperation between ERα and GPER in ovarian cancer cells.

Although these data may appear contradictory, it is not surprising that the intricate network of ligand-activated cell responses could lead to divergent biological outcomes, as discussed above. In this regard, well-designed assays have recently shown how the differential engagement of feedback and feed-forward regulation by different ligands leads to different dynamics of pathway activity, which in turn alters cell fate (Ryu et al. 2015). One plausible network motif that drives these responses at least in part may be a transient, pulsing, or prolonged activation of certain transduction pathway(s) beyond a threshold level. Hence, different signaling frequencies and amplitudes could uncover the timescales of major network components determining ultimate cell choices (Purvis and Lahav 2013). As Chevalier et al. suggest, we need to boost research and innovative tools to better appreciate the multifaceted mechanisms of action and the biological effects of environmental contaminants on human health.
